# Antibiotic-resistant bacteria, antibiotic resistance genes, and antibiotic residues in wastewater from a poultry slaughterhouse after conventional and advanced treatments

**DOI:** 10.1038/s41598-021-96169-y

**Published:** 2021-08-17

**Authors:** Mykhailo Savin, Johannes Alexander, Gabriele Bierbaum, Jens Andre Hammerl, Norman Hembach, Thomas Schwartz, Ricarda Maria Schmithausen, Esther Sib, Alexander Voigt, Judith Kreyenschmidt

**Affiliations:** 1 Institute for Hygiene and Public Health, Medical Faculty, University of Bonn, Venusberg-Campus 1, 53127 Bonn, Germany; 2 Institute of Animal Sciences, University of Bonn, Bonn, Germany; 3 Department of Microbiology/Molecular Biology, Institute of Functional Interfaces (IFG), Karlsruhe Institute of Technology, Eggenstein-Leopoldshafen, Germany; 4 Institute for Medical Microbiology, Immunology and Parasitology, Medical Faculty, University of Bonn, Bonn, Germany; 5 Department for Biological Safety, German Federal Institute for Risk Assessment, Berlin, Germany; 6 Department of Fresh Produce Logistics, Hochschule Geisenheim University, Geisenheim, Germany

**Keywords:** Applied microbiology, Environmental microbiology

## Abstract

Slaughterhouse wastewater is considered a reservoir for antibiotic-resistant bacteria and antibiotic residues, which are not sufficiently removed by conventional treatment processes. This study focuses on the occurrence of ESKAPE bacteria (*Enterococcus* spp., *S. aureus*, *K. pneumoniae*, *A. baumannii*, *P. aeruginosa*, *Enterobacter* spp.), ESBL (extended-spectrum β-lactamase)-producing *E. coli*, antibiotic resistance genes (ARGs) and antibiotic residues in wastewater from a poultry slaughterhouse. The efficacy of conventional and advanced treatments (i.e., ozonation) of the in-house wastewater treatment plant regarding their removal was also evaluated. Target culturable bacteria were detected only in the influent and effluent after conventional treatment. High abundances of genes (e.g., *bla*_TEM_, *bla*_CTX-M-15_, *bla*_CTX-M-32_, *bla*_OXA-48_, *bla*_CMY_ and *mcr-1*) of up to 1.48 × 10^6^ copies/100 mL were detected in raw influent. All of them were already significantly reduced by 1–4.2 log units after conventional treatment. Following ozonation, *mcr-1* and *bla*_CTX-M-32_ were further reduced below the limit of detection. Antibiotic residues were detected in 55.6% (n = 10/18) of the wastewater samples. Despite the significant reduction through conventional and advanced treatments, effluents still exhibited high concentrations of some ARGs (e.g., *sul1*, *ermB* and *bla*_OXA-48_), ranging from 1.75 × 10^2^ to 3.44 × 10^3^ copies/100 mL. Thus, a combination of oxidative, adsorptive and membrane-based technologies should be considered.

## Introduction

Currently, poultry represents one of the main sources of meat production worldwide^1^. In the first half of 2020, more than 800,000 tons of poultry were processed only in Germany^2^. Slaughtering, processing and cleaning and sanitizing of poultry production facilities are water-consuming processes. Depending on the slaughtering process, between 5,000 and 21,000 L of water per ton of meat is needed for processing^3^. This leads to high amounts of wastewater that arises along the slaughtering chain, which usually exhibits various enteric pathogens^4^. In addition to microbiological loads, slaughterhouse wastewater also exhibits a high organic content due to solved fibers, proteins, and fats^5^. Thus, the direct discharge of untreated livestock wastewater to surface waters is impractical and should be avoided because of environmental pollution and possible negative effects on human health. Moreover, after the prescribed withdrawal periods, it may further contain residues of antimicrobials commonly used in veterinary medicine as well as detergents and disinfectants^6,7^.

Interestingly, meat production has changed over the last 30 years, as it has doubled worldwide and is expected to double again until 2050^8^. This implies a steadily increasing quantity of produced wastewater, which also forces an increase in the pollution loads discharged into the environment. Thus, mostly after pretreatment in on-site wastewater treatment plants (WWTPs), slaughterhouses commonly discharge their wastewater either directly into a river or other receiving body (direct dischargers) or to municipal WWTPs (indirect dischargers)^9,10^.

German slaughterhouses commonly treat their wastewater by different physicochemical and/or biological methods. This results in a moderate to high removal of nutrients and a reduction of the bacterial load by 1.1–3.4 log units^6,11,12^. Currently, advanced oxidation processes (AOPs) are becoming an interesting additional treatment option to the prevailing conventional methods. AOPs (e.g., ozone treatment) are currently discussed as effective technologies for inactivation of microorganisms, especially antibiotic-resistant bacteria and pathogens^12^. Ozone treatment has been shown to exhibit a high reduction efficacy of 98.4% against facultative pathogenic bacteria and their antibiotic resistance genes (ARGs)^12^. In German municipal WWTPs, microbial reduction to below the detection limit (10^1^ cell equivalents per 100 mL) was recently reported in the case of additional advanced treatment technologies (ozonation and ultrafiltration)^13^. Thus, advanced treatment technologies could decrease the bacterial loads discharged into the aquatic environment, preventing the dissemination, inter alia, of clinically relevant antibiotic-resistant bacteria and their resistance determinants. However, upgrading WWTPs with advanced treatment technologies bears additional costs associated with high energy consumption, additional personnel and posttreatment of the effluents^12,14^.

Interestingly, in the EU, the use of advanced treatment technologies is not mandatory for operators of WWTPs in any sector (e.g., municipal, health care or industry), as no legal limits or reduction levels have been fitted for microbiological pollutants in wastewater. However, the occurrence of important pathogenic microorganisms in municipal and clinical wastewater is well documented^15^. Furthermore, antibiotic-resistant bacteria with zoonotic potential are prevalent in livestock wastewater^16^. Livestock-associated methicillin-resistant *Staphylococcus aureus* (LA-MRSA), extended-spectrum β-lactamase (ESBL)-producing *Escherichia coli/Klebsiella pneumoniae* and *mcr-1-*carrying Enterobacterales, exhibiting resistance to highly and critically important antimicrobials, were already detected in biologically and physicochemically treated effluents of in-house WWTPs of German poultry slaughterhouses^17,18^. In addition, Savin et al. reported the detection of antimicrobial residues in wastewater from German pig slaughterhouses after conventional treatment in their on-site WWTPs^19^. This provides evidence on inadequate wastewater treatment by in-house WWTPs that enables further dissemination of clinically relevant bacteria of livestock origin and antimicrobial residues into surface waters and the environment.

However, comparable data on the occurrence of antibiotic-resistant facultative pathogenic bacteria and antimicrobial residues in livestock wastewater before and after treatment by advanced techniques such as ozonation are lacking. This study aimed to determine the occurrence of ESKAPE bacteria (*Enterococcus* spp., *S. aureus*, *K. pneumoniae*, *Acinetobacter baumannii*, *Pseudomonas aeruginosa*, *Enterobacter* spp.), ESBL-producing *E. coli* and antimicrobial residues in wastewater from a poultry slaughterhouse before and after conventional and advanced treatments. Furthermore, the reduction efficacy of the in-house WWTP upgraded with an ozone facility on clinically relevant ARGs and facultative pathogenic bacteria (i.e., *E. coli*, *K. pneumoniae*, *A. baumannii*, *P. aeruginosa* and enterococci) was evaluated.

## Results

### Detection of target ESKAPE bacteria and their phenotypic resistance

Overall, 97 isolates were recovered from water samples of the influent (n = 81) and effluent after physicochemical and biological treatments (n = 16). No target ESKAPE bacteria were detected in the effluent after subsequent ozone treatment. The majority of them belonged to the *A. calcoaceticus-baumannii* complex (ACB complex: 32.0%, 31/97), *K. pneumoniae* (24.7%, 24/97), *E. coli* (22.7%, 22/97) and *S. aureus* (14.4%, 14/97). The abundances of the *E. cloacae* complex and *P. aeruginosa* were low at 4.1% (4/97) and 2.1% (2/97), respectively. No VRE and CRE were detected.

**Figure 1:**
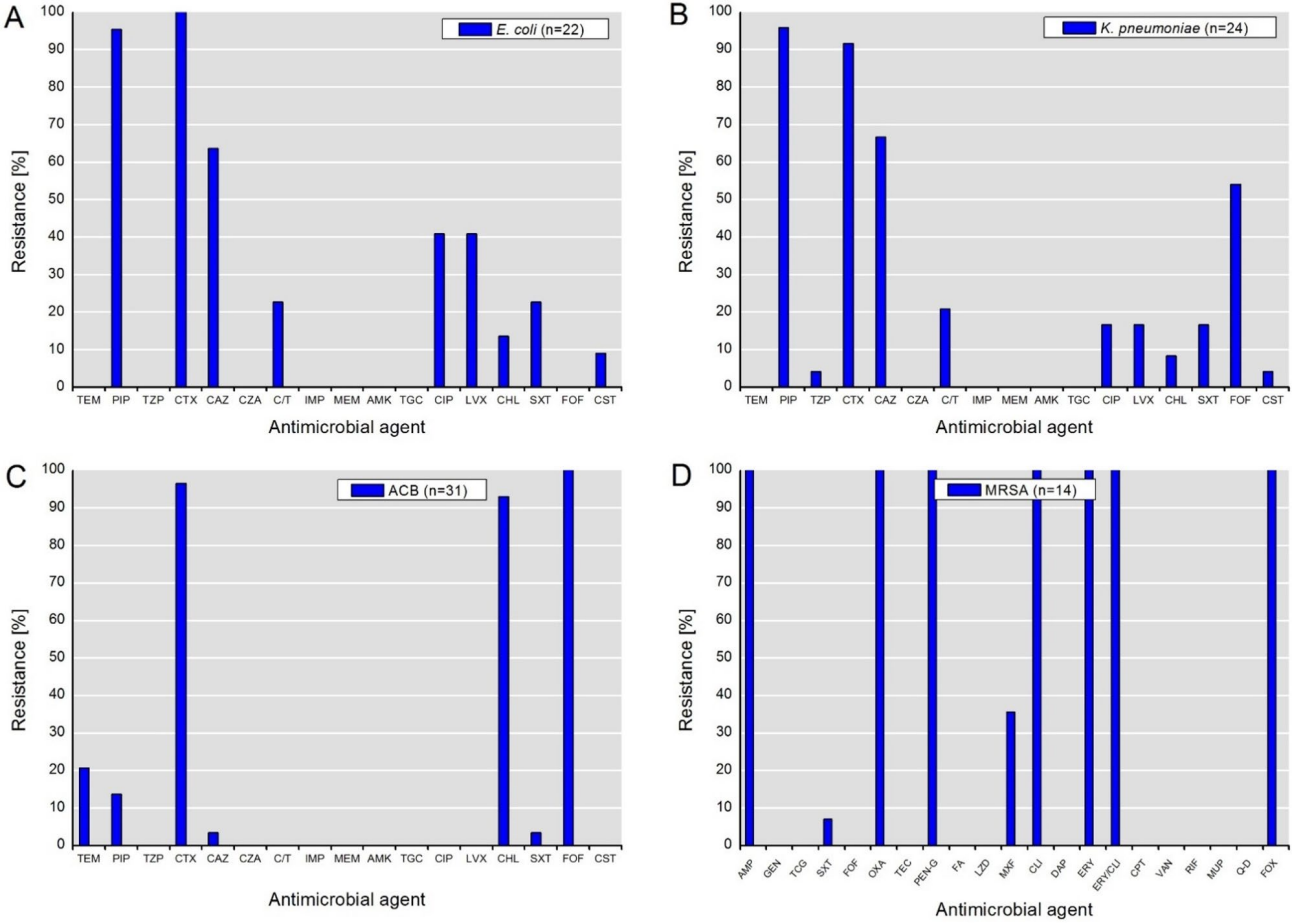
Phenotypic resistance to antimicrobial agents detected among isolates of (**A**) *E. coli*, (**B**) *K. pneumoniae*, (C*) ACB complex and (D**) MRSA. Abbreviations for antimicrobial agents: *TEM* temocillin, *PIP* piperacillin, *TZP* piperacillin-tazobactam, *CTX* cefotaxime; *CAZ* ceftazidime; *CZA* ceftazidime-avibactam; *C/T* ceftolozane-tazobactam; *IMP* imipenem, *MEM* meropenem, *AMK* amikacin, *TGC* tigecycline, *CIP* ciprofloxacin, *LVX* levofloxacin, *CHL* chloramphenicol, *SXT* sulfamethoxazole-trimethoprim, *FOF* fosfomycin, *CST* colistin, *AMP* ampicillin, *GEN* gentamicin; *OXA* oxacillin, *TEC* teicoplanin, *PEN-G* penicillin G, *FA* fusidic acid, *LZD* linezolid, *DAP* daptomycin, *CPT* ceftaroline,, *VAN* vancomycin, *RIF* rifampicin, *MUP* mupirocin, *FOX* cefoxitin; *MXF* moxifloxacin, *CLI* clindamycin; *ERY* erythromycin, *Q-D* synercid (quinupristin-dalfopristin). *Species of the ACB complex are considered intrinsically resistant to temocillin, cefotaxime, chloramphenicol and fosfomycin. **MRSA is considered intrinsically resistant to ampicillin, oxacillin, penicillin-G and cefoxitin.

The phenotypic antimicrobial resistance of the recovered isolates is summarized in Fig. 1. Due to selective isolation, high rates of resistance to β-lactams (e.g., PIP and CTX) among isolates of *E. coli* and *K. pneumoniae* were expected. The highest 3MDRO rate (multidrug-resistant organisms) with combined resistance to PIP, CTX and CIP was exhibited by *E. coli* (40.9%), followed by *K. pneumoniae* (16.7%). Notably, all isolates were susceptible to carbapenems (IMP, MEM), ceftazidime-avibactam, amikacin and tigecycline. Interestingly, 22.7% and 20.8% of *E. coli* and *K. pneumoniae* isolates, respectively, exhibited resistance to the newly approved drug combination ceftolozane-tazobactam. Resistance to colistin was detected only at a low rate among *E. coli* (9.1%) and *K. pneumoniae* (4.2%) isolates. Furthermore, no 3MDRO phenotype was detected among isolates of the ACB complex. Moreover, considering the intrinsic resistance of ACB complex species to temocillin, cefotaxime, chloramphenicol and fosfomycin, only a minor percentage of isolates exhibited acquired resistance to ceftazidime and sulfamethoxazole-trimethoprim. Interestingly, in addition to the intrinsic resistance to ampicillin, oxacillin, penicillin-G, and cefoxitin, all MRSA isolates were resistant to clindamycin, erythromycin and the combination thereof.

### Characterization of ARGs in target bacterial species

The majority of *E. coli* isolates (72.7%, 16/22) exhibited a *bla*_TEM_ genotype, with *bla*_TEM-116_ accounting for 27.3%, as well as *bla*_TEM-52_ and *bla*_TEM-1_ accounting for 22.7% each, respectively. *bla*_CTX-M-1_ and *bla*_SHV-12_ were detected in 13.6% of *E. coli* isolates. In *K. pneumoniae* (n = 24), only genes of the *bla*_SHV_ family were identified, with *bla*_SHV-2_ accounting for 66.7% and *bla*_SHV-12_ accounting for 8.3% of the isolates. Six *K. pneumoniae* isolates (25.0%) tested negative for *bla* genes encoding SHV, TEM, and CTX-M enzymes. Isolates of the *E. cloacae* complex (n = 4) carried *bla*_SHV-12_ (n = 2) and *bla*_TEM-1_ (n = 2).

No *bla*_PER_, *bla*_GES_, or *bla*_VEB_ were detected among *P. aeruginosa* and isolates of the ACB complex. *mcr-1.1* was detected in one colistin-resistant *E. coli* (1/2) isolate.

### Molecular typing of ESBL-producing *E. coli* and MRSA

The majority of *E. coli* isolates were assigned to phylogroups B1 (50.0%) and E (27.3%), which are commonly associated with commensal strains. Less abundant phylogroups were represented by A and F (each 9.1%) as well as C (4.5%). Notably, extraintestinal pathogenic (ExPEC) groups B2 and D were not detected.

Recovered MRSA isolates (n = 14) belonged to the clonal complexes CC9 (t1430 and t13177, each 35.7%) and CC398 (t034, 28.6%).

### Abundance of ARGs in water samples

**Figure 2:**
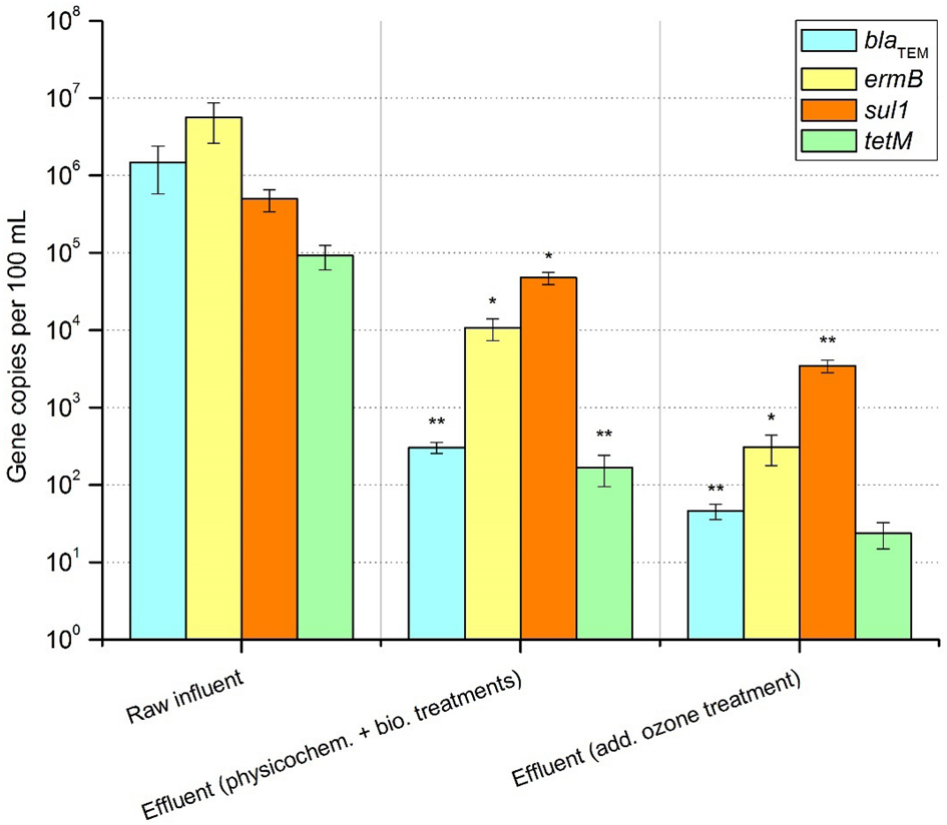
Abundance of “frequent” antibiotic resistance genes in influent and effluent after physicochemical and biological treatments and subsequent ozone treatment. Displayed are mean values with standard deviation. Significance is given by one-tailed nonparametric Mann–Whitney U test calculation and is shown by asterisks (*p ≤ 0.05, **p ≤ 0.001). Compared are gene copies per 100 mL in (i) raw influent with effluent after physicochemical and biological treatments and (ii) in effluent after physicochemical and biological treatments with effluent after subsequent ozone treatment.

According to Hembach et al.^13^, the abundance of “frequent” ARGs in influent, effluent after physicochemical and biological treatments, and subsequent ozone treatment is shown in Fig. 2. All measurements were performed after PMA treatment for living/dead discrimination. *ermB* and *bla*_TEM_ exhibited the highest abundances of 5.61 × 10^6^ and 1.48 × 10^6^ gene copies per 100 mL, respectively, followed by *sul1* (4.98 × 10^5^) and *tetM* (9.25 × 10^4^). After physicochemical and biological treatments, the highest significant reduction of 3.7 log units was achieved for *bla*_TEM_ (*p* < 0.001), followed by *ermB* and *tetM* (each 2.7 log units) as well as *sul1* (1 log unit). After the subsequent ozonation, *bla*_TEM_, *sul1* (both *p* < 0.001) and *ermB* (*p* < 0.05) were further significantly reduced (6.6 – 34.4-fold).

**Figure 3:**
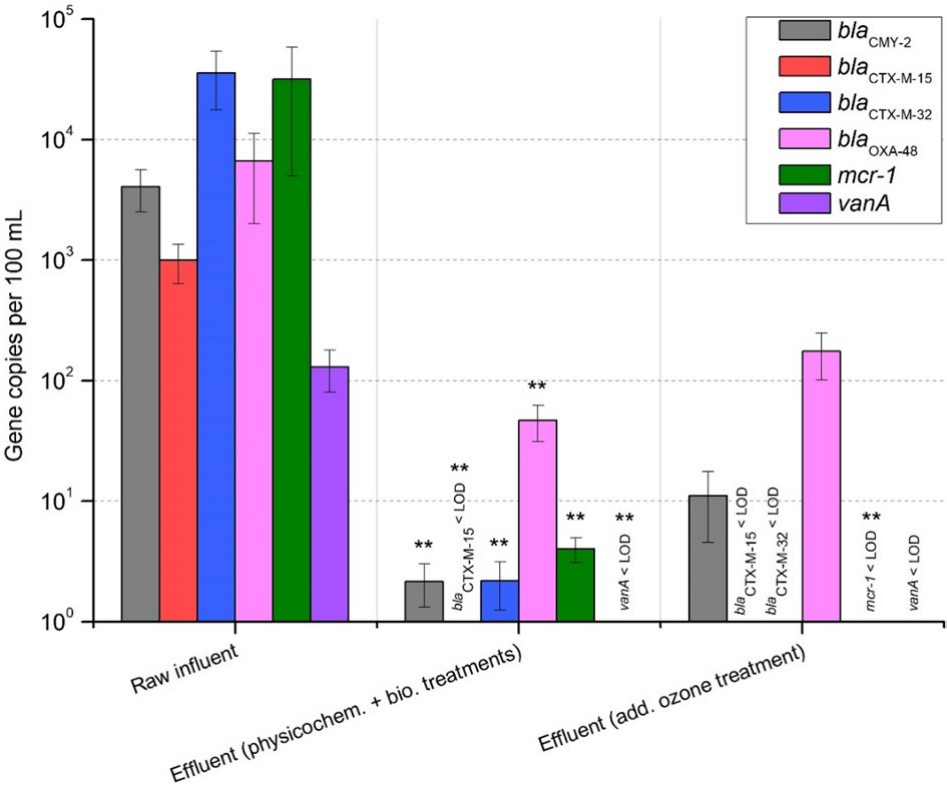
Abundance of “intermediate and rare abundant” antibiotic resistance genes in influent and effluent after physicochemical and biological treatments and subsequent ozone treatment. Displayed are mean values with standard deviation. Significance is given by one-tailed nonparametric Mann–Whitney U test calculation and is shown by asterisks (**p ≤ 0.001). Compared are gene copies per 100 mL in (i) raw influent with effluent after physicochemical and biological treatments and (ii) in effluent after physicochemical and biological treatments with effluent after subsequent ozone treatment. LOD – limit of detection.

The abundance of “intermediate and rare abundant” ARGs in the analysed samples is shown in Fig. 3. *bla*_CTX-M-32_ and *mcr-1* were detected with the highest abundances of 3.58 × 10^4^ and 3.17 × 10^4^ gene copies per 100 mL, respectively, followed by *bla*_OXA-48_ (6.65 × 10^3^), *bla*_CMY-2_ (4.07 × 10^3^), *bla*_CTX-M-15_ (9.97 × 10^2^), and *vanA* (1.30 × 10^2^) gene copies per 100 mL. However, all of them were significantly reduced after physicochemical and biological treatments (*p* < 0.001), with reduction factors ranging from 2.2 log units in the case of carbapenemase *bla*_OXA-48_ to 4.2 log units in the case of *bla*_CTX-M-32_. Furthermore, *bla*_CTX-M-15_ and *vanA* were reduced to under the limit of detection (LOD). After subsequent ozonation, *bla*_CTX-M-32_ (*p* < 0.05) and *mcr-1* (*p* < 0.001) were significantly reduced < LOD.

### Abundance of facultative pathogenic bacteria in water samples

**Figure 4:**
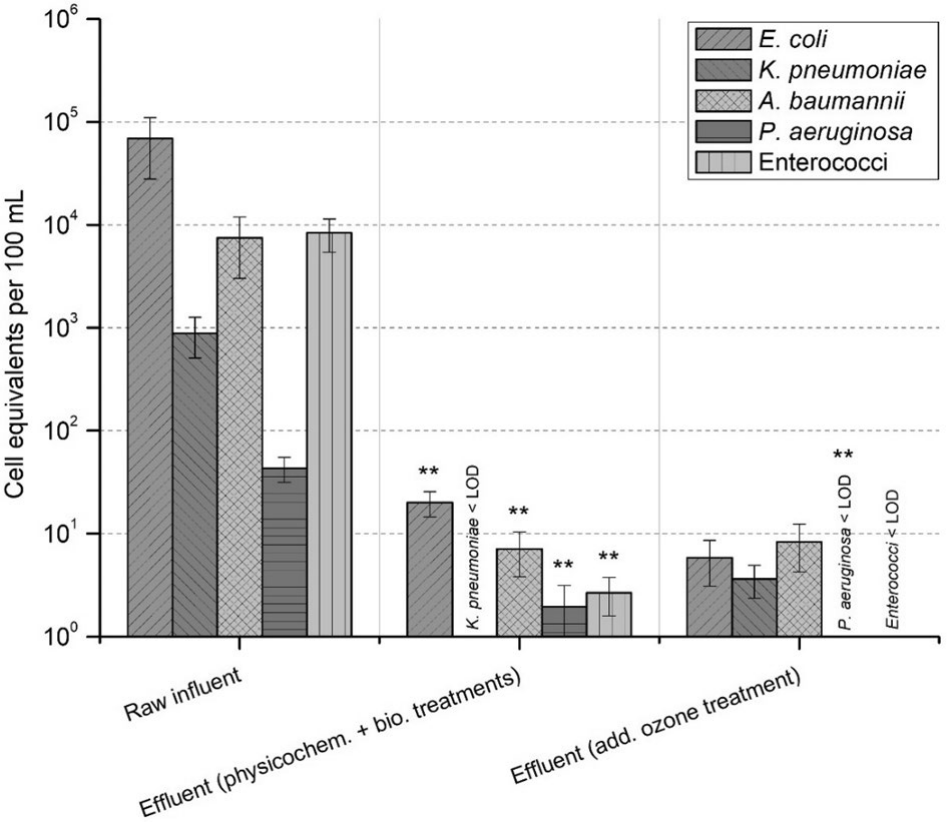
Abundance of facultative pathogenic bacteria in influent and effluent after physicochemical and biological treatments and subsequent ozone treatment. Displayed are mean values with standard deviation. Significance is given by one-tailed nonparametric Mann–Whitney U test calculation and is shown by asterisks (**p ≤ 0.001). Compared are gene copies per 100 mL in (i) raw influent with effluent after physicochemical and biological treatments and (ii) in effluent after physicochemical and biological treatments with effluent after subsequent ozone treatment. LOD – limit of detection.

*E. coli*, *K. pneumoniae*, *A. baumannii*, *P. aeruginosa,* and enterococci were quantified using species-specific gene markers after PMA treatment of the samples (Fig. 4). *E. coli* followed by enterococci and *A. baumannii* were the most predominant target species in the influent, with abundances of 6.90 × 10^4^, 8.41 × 10^3^ and 7.47 × 10^3^ cell equivalents per 100 mL, respectively. *P. aeruginosa* exhibited the lowest concentration of 4.32 × 10^1^ cell equivalents per 100 mL.

After the physicochemical and biological treatments, all target species were reduced significantly (*p* < 0.001). Moreover, *K. pneumoniae* was reduced < LOD. After the subsequent ozonation, a nonsignificant reduction in *E. coli* and enterococci was observed, whereas *P. aeruginosa* was significantly reduced < LOD (*p* < 0.001).

### Occurrence of antimicrobial residues in water samples

Of the wastewater samples, 55.6% (n = 10/18) were positive for antibiotic residues, which were detected only in the influent and effluent of the in-house WWTP after conventional treatment, indicating that antimicrobials were removed during ozonation (Table 1). The most prevalent antimicrobial classes detected were macrolides (38.9%, 7/18), sulfonamides (16.7%, 3/18), and fluoroquinolones (11.1%, 2/18).

**Table 1:** Antimicrobial residues detected in wastewater samples from the in-house WWTP of the examined poultry slaughterhouse.

Antimicrobial^a^ [µg/L]	Influent (n = 6)					Effluent after physicochemical and biological treatments (n = 6)					Frequency ^c^[%]
	N^b^1	N2	N3	N4	N6	N1	N2	N3	N4	N5	
TYL	3.7	0.83			12	0.27	0.24	0.08		0.11	38.9
SXM				0.06					0.04		11.1
SDD									0.06		5.6
ENR			3.9		0.47						11.1
AMP	< LOQ										-

^a^Abbreviations for antimicrobial agents: *TYL* tylosin, *SXM* sulfamethoxazole, *SDD* sulfadimidine, *ENR* enrofloxacin, *AMP* ampicillin.

^b^N—Sampling number.

^c^Only samples with concentrations > LOQ were used for the evaluation.

## Discussion

This study provides insights regarding the occurrence and impact of ESKAPE bacteria, including ESBL-producing *E. coli*, ARGs and antimicrobial residues, in wastewater samples from a German poultry slaughterhouse that might be disseminated to the environment. Furthermore, we evaluated the efficacy of ozonation treatment of wastewater for a reduction in bacterial loads and ARGs.

The high number of notified 3MDRO *E. coli* and *K. pneumoniae* isolates might be caused by the use of β-lactams (e.g., cephalosporines, penicillins) and fluoroquinolones for the treatment of animal infections in the veterinary medicine^20^. Cephalosporines and fluoroquinolones belong to the veterinary critically important antimicrobial agents (VCIAs) and are crucial for combating specific infectious diseases (e.g., septicaemias and respiratory and enteric diseases) in livestock. However, the use of VCIA also affects public health, as these antimicrobials are also considered the highest priority critically important antimicrobials (HPCIAs) for humans^21^.

Clinically associated fluoroquinolone resistance is mainly caused by chromosomal mutations in the quinolone resistance-determining regions (QRDRs) of *gyrA* and *parC*^22,23^. Furthermore, the cooccurrence of plasmid-mediated quinolone resistance (PMQR) genes and QRDR mutations can lead to high resistance (fluoro)quinolone phenotypes^24^. Recently, high percentages of ciprofloxacin resistance in ESBL-producing *E. coli* (50.5%) and *K. pneumoniae* (67.9%) from process and wastewater of poultry slaughterhouses have been reported^17^. Moreover, Savin et al. also notified a high number of PMQRs (31.4%) in 3MDRO extraintestinal pathogenic *E. coli* (ExPEC) and ESBL-producing *K. pneumoniae* (100%) from poultry slaughterhouses^16^.

The detected concentrations of enrofloxacin in untreated wastewater exceeded its Predicted No Effect Concentrations (PNECs) for resistance selection of 0.064 µg/L^25^, indicating that the withdrawal period of enrofloxacin might not be strictly adhered to. This selective pressure might further facilitate the development of fluoroquinolone resistance in clinically relevant bacteria. Thus, the use of fluoroquinolones should be restricted to individual treatments and no longer permitted for general medication of poultry herds. Thus, we strongly recommend forcing the development of alternative antimicrobial-free treatment strategies to avoid the use of similar antimicrobials in different compartments.

The high abundance of β-lactam (*bla*) resistance genes in the investigated cephalosporin-resistant *E. coli* correlates well with the detected prevalence of *bla*_TEM-1_, *bla*_TEM-52_, *bla*_TEM-116_, *bla*_SHV-1_, *bla*_SHV-12_ and *bla*_CTX-M-1_ in isolates of livestock wastewater and poultry products^26,27^. In general, ESBL-producing *E. coli* carrying diverse virulence determinants (i.e. for human colonization and infection) are widely disseminated in German poultry production^16,28^. While other reports have detected up to 16.2% ExPEC isolates from poultry wastewater, no isolate of this study was assigned to the ExPEC phylogroups B2 or D^16^.

The development and dissemination of ESBL-producing *Enterobacteriaceae* are not only promoted by the use of β-lactams in veterinary medicine but also facilitated by co- and cross-resistance through disinfectants, heavy metals, and other antimicrobials, since corresponding genes are often located on the same plasmid^29^. The cooccurrence of *bla* and *mcr* genes is of particular importance, since colistin was reintroduced into human therapy to treat infections caused by carbapenemase-producing *Enterobacteriaceae* (CPE) or multidrug-resistant *A. baumannii* and *P. aeruginosa*^30^. Moreover, mobile colistin resistance genes (esp. *mcr-1*) is considered to originate from livestock^31^. Colistin-resistant *Enterobacteriaceae* carrying *mcr-1* on a wide variety of transmissible plasmids have already been reported from German process waters and wastewaters of livestock slaughterhouses, municipal WWTPs, and hospitals^18,32^.

Reports on the occurrence of ARGs in livestock wastewater conferring resistance to HPCIA have already been published^33^. However, there is a lack of qualitative and quantitative data for German slaughterhouses. We detected high abundances of the *mcr-1*, *bla*_CMY-2_ and *bla*_CTX-M_ genes in untreated wastewater, which is worrying since they confer resistance to HPCIA in humans^20^. Their potential incorporation into clinically relevant bacteria might narrow antimicrobial treatment options and compromise their efficacy in cases of infection, especially if resistance against different last resort antibiotics (e.g., carbapenemases, *mcr*) occurs^34,35^. The human impact of livestock as a reservoir for combinations of antimicrobials of the last resort (i.e. *bla*_NDM-1_/*mcr-1*) have already been described^36,37^.

Of special concern is the occurrence of the carbapenemase gene *bla*_OXA-48_ among investigated wastewater samples detected by molecular-based methods. As selective cultivation did not yield any carbapenem-resistant isolates, culturable carbapenemase-producing *Enterobacteriaceae* can be excluded as predominant hosts for *bla*_OXA-48_. Thus, molecular detection of carbapenemase genes in environmental samples has only limited value, as the host species is not identified. However, other studies demonstrated different transferable plasmids carrying the *bla*_OXA-48_ gene in *Enterobacteriaceae*, which might not be cultivable or underrepresented in the analysed isolates of our study^38,39^. A probable reservoir for *bla*_OXA-48_ might be *Shewanella* spp., as this gene naturally occurs in these bacteria. As *Shewanella* spp. are not of particular clinical relevance, they might play a role as drivers of *bla*_OXA-48_ spread to clinically relevant bacteria (i.e. ESKAPE)^40^. The slight increase of *bla*_OXA-48_ in effluent samples treated with zone might indicate its selective properties regarding antibiotic-resistant, and more ozone-tolerant bacterial species^41^.

The presence of the *vanA* gene in untreated wastewater is worrying, as vancomycin also represents an antimicrobial of the last resort that is critically important for human medicine^21^. However, cultural analysis for vancomycin-resistant enterococci (VRE) was negative, which is in line with other studies reporting the absence or rare occurrence of VRE in livestock wastewater from German slaughterhouses^17,19^. This might indicate the presence of vancomycin-variable enterococci (VVE), which are *vanA*-positive but phenotypically susceptible to vancomycin^42^. Because of their susceptibility to vancomycin, traditional methods fail to detect VVE. *vanA* encodes inducible high-level resistance to glycopeptides, and VVE have the ability to revert into vancomycin-resistant phenotypes upon vancomycin exposure^42^. However, glycopeptides are not approved for the treatment of livestock in the EU, and the use of avoparcin in feed as a growth promoter was banned in Germany in 1996^43^. Moreover, after treatments in WWTPs, *vanA* and enterococci were reduced below the detection limit. Thus, the role of agriculture in Germany in the development of glycopeptide-resistant enterococci is less significant than that in the general community and hospitals. *Pseudomonas* spp., *Aeromonas* spp. and *Raoutella* spp. isolated from surface water have also been reported to carry the *vanA* gene^44^.

However, coselection by macrolides, tetracycline, or copper might play an important role in maintaining the persistence of VRE in livestock^45–47^. Macrolides and tetracyclines together with penicillins and sulfonamides belong to the VCIA, for which there are fewer alternatives available^48^. In 2018, 571.7 tons of antimicrobials of these classes were sold to veterinarians in Germany, making up 79.1% of the total amount^49^. Interestingly, the supplied quantities of these antimicrobials correlate well with the abundances of particular ARGs in the investigated wastewater samples. Moreover, high abundances of *ermB*, *sul1* and *tetM* genes are in line with other studies reporting a high prevalence of these resistance determinants in isolates recovered from livestock wastewater and poultry products, inter alia, in ExPEC and MDR *E. coli* isolates^16^.

The high abundance of *ermB* in wastewater is critical since *ermB* encodes resistance to macrolides, which are HPCIAs for human medicine and are crucial for the treatment of severe *Campylobacter* infections, particularly in children^21^. Furthermore, *erm* genes could be transferred to gram-positive pathogens (e.g., staphylococci, streptococci, enterococci), resulting in MLS_B_ (macrolide, lincosamide, streptogramin B) cross-resistance, compromising the efficacy of further antibiotics such as erythromycin and clindamycin^50^. This might be the reason for the high resistance rates of MRSA to erythromycin and its combination with clindamycin. MRSA of CC9 and CC398, which were detected in this study, have already been reported in wastewater from poultry slaughterhouses and in retail poultry products^17,51^. They were also isolated from human infections, especially in regions with high livestock production, underlying their zoonotic potential^52^. Interestingly, the detected concentration of tylosin (12 µg/L) might exert selective pressure on species with resistance to macrolides, including MRSA, as it exceeded its PNEC of 4 µg/L. However, detected antimicrobials were eliminated after ozonation, which is in line with other studies reporting high degradation rates (70–100%) of macrolides as well as sulfonamides and fluoroquinolones depending on ozone dose and reaction time^53^. The removal of various organic micropollutants, which might not be reduced by other advanced treatment technologies, e.g., ultrafiltration, is an advantage of ozonation.

However, additional ozonation following conventional treatment did not significantly reduce the loads of facultative pathogenic bacteria, which might be due to different factors, such as ozonation time, concentration of the applied ozone, wastewater composition and physical characteristics of the examined organisms^41^. It is important to note that a high number of bacterial cells in wastewater might be aggregated in flocs, protecting the inner cells from the damaging effects of ozone. Comparable results regarding microbial reduction rates were observed in German municipal WWTPs after conventional treatment^54^.

Conventional treatment significantly reduced the abundances of ARGs conferring resistance to clinically relevant antimicrobials. Some of them (i.e., *mcr-1*, *bla*_CTX-M-32_) were further reduced below the limit of detection after subsequent additional treatment with ozone, underlying the importance of advanced treatment. However, despite the significant reduction through conventional and advanced treatments, effluents still exhibited relatively high concentrations of some ARGs, e.g., *sul1*, *ermB* and *bla*_OXA-48_ (> 10^2^ copies/100 mL). Similar reduction rates were reported by Czekalski and colleagues^41^. Ozonation does not show the best effect on the reduction of ARGs in comparison to other methods, e.g., chlorination or ultrafiltration. This might be due to low ozone dosages frequently used in practice^13,55^. However, increasing the amount of ozone might lead to the release of harmful products to the environment and have an ecotoxicological effect^56^. Applying additional filtration steps (e.g., charcoal or sand filtration) after ozonation to remove remaining ozone and unwanted byproducts may result in bacterial regrowth^13^. Moreover, the receiving water bodies can support bacterial regrowth if they contain the necessary nutrients. Iakovides et al. reported the regrowth of total and antibiotic-resistant *E. coli* after the stress caused by ozone was relieved, indicating the importance of proper setting of ozonation parameters^53^. For wastewater exhibiting high concentrations of ARGs and antibiotic-resistant bacteria, a combination of oxidative, adsorptive (charcoal or sand filtration), and membrane-based technologies should be considered to interrupt dissemination of (facultative) pathogenic antibiotic-resistant bacteria to the environment.

## Material and methods

### Examined poultry slaughterhouse and its wastewater management

The investigated poultry slaughterhouse exhibited a capacity of > 100,000 slaughtered chickens per day by producing 3,600 m^3^ wastewater that was treated in an in-house wastewater treatment plant (WWTP) before being discharged into a preflooder and a receiving waterbody (i.e., river).

On-site treatment of wastewater is based on physicochemical, biological, and advanced oxidation processes. First, the wastewater was mechanically pretreated using screeners and sieves. Fat and greases are removed using grease traps. Afterwards, the wastewater is treated by dissolved air flotation with a subsequent biological treatment by activated sludge. Additionally, the in-house WWTP was equipped with an ozone system (Xylem Water Solutions Deutschland GmbH, Großostheim, Germany). The ozone dosage used was 75 g/m^3^, and the contact time varied between 15 and 30 min, depending on the water flow rate.

### Sampling and sample preparation

In a two-month period, the in-house WWTP of a poultry slaughterhouse was sampled six times with a minimum time interval of 1 week. During sampling, a total of 18 samples were collected representing the influent (n = 6), the effluent after physicochemical and biological treatments (n = 6) and the effluent after ozone treatment (n = 6). The samples were taken as qualified samples according to the German standard methods for the examination of water, wastewater, and sludge (DIN 38402-11:2009-02)^57^. Therefore, five subsamples of 200 mL in two-minute sampling intervals were collected and mixed. The samples were transported to the laboratory in a Styrofoam box cooled to 5 ± 2 °C and were further processed within 24 h. Influent samples were manually filtered using stomacher strainer bags with a tissue filter (pore size, 0.5 mm; VWR, Radnor, PA, USA) to remove large particles.

### Cultivation, identification and susceptibility testing of target bacteria

The samples were subjected to culturing on selective media to isolate gram-negative ESBL-producing and carbapenemase-producing *Enterobacteriaceae* (*E. coli*, *Klebsiella* spp., *Enterobacter* spp., *Citrobacter* spp. ), nonfermenting *A. baumannii* and *P. aeruginosa*, as well as MRSA and vancomycin-resistant enterococci (VRE). Detailed information on their cultivation procedures has already been published^17^.

Species identification was performed by MALDI-ToF MS (bioMérieux, Marcy-l'Étoile, France) equipped with Myla software. The isolates were purified on Columbia agar supplemented with 5% sheep blood, and cryopreservation at − 80 °C in cryotubes (Mast Diagnostics, Reinfeld, Germany) was used for storage. Isolated target bacteria were further subjected to antimicrobial susceptibility testing by the microdilution method according to protocols of the European Committee on Antimicrobial Susceptibility Testing (EUCAST v 11.0) using the Micronaut-S MDR MRGN-Screening system for Gram-negatives and the MICRONAUT-S MRSA/GP testing panel for Gram-positive bacteria (MERLIN, Gesellschaft für mikrobiologische Diagnostika GmbH, Bornheim-Hersel, Germany)^58^. The results were evaluated based on clinical cut-off values provided by EUCAST^59^. The multidrug-resistance phenotype (3MDRO) was defined based on combined resistance to piperacillin (PIP), cefotaxime (CTX) and/or ceftazidime (CAZ), and ciprofloxacin (CIP) as previously described^60^.

### Detection and analysis of selected antibiotic resistance genes (ARGs) in target bacteria

Template DNA for PCRs was prepared by boiling bacterial suspensions in 10 mM Tris–EDTA pH 8.0 (Sigma-Aldrich, St. Louis, MO, USA) according to Aldous et al.^61^. Enterobacterales with phenotypic resistance to 3rd-generation cephalosporins were screened by PCR for β-lactamase (*bla*) genes encoding enzymes SHV, TEM, and CTX-M (groups 1, 2, 8 and 9) as previously described^62–64^. Isolates of the *A. calcoaceticus-baumannii* complex and *P. aeruginosa* were examined for the presence of *bla*_PER_, *bla*_GES_, and *bla*_VEB_ by PCR as described^65^. Colistin-resistant isolates (MIC > 2 mg/L) were screened for *mcr-1* to *mcr-5* as well as *mcr-6* to *mcr-9* genes using multiplex PCR protocols as described by Rebelo et al.^66^ and Borowiak et al.^67^, respectively. The obtained PCR amplicons were purified with the innuPREP DOUBLEpure Kit (Analytik Jena AG, Jena, Germany) and subjected to Sanger sequencing at Microsynth Seqlab (Göttingen, Germany).

### Molecular typing of resistant bacterial isolates

Phylotyping of *E. coli* isolates (A, B1, B2, C, D, E, F, clade I-V) was conducted as previously described^68^. MRSA isolates were *spa*-typed by amplifying and sequencing the *Staphylococcus protein* A repeat region according to Harmsen et al.^69^. The Ridom *spa* server database (http://www.spaserver.ridom.de) was used for assignment of *spa* types.

### DNA extraction from water samples, quantification of antibiotic resistance genes (ARGs) and taxonomic gene markers

Volumes of 50 mL, 200 mL and 400 mL of influent, effluent after physicochemical and biological treatments and effluent after ozone treatment were subjected to DNA isolation after treatment with 0.25 mM propidium monoazide (PMA) (BLU-V viability PMA-kit, Qiagen GmbH, Hilden, Germany) as previously described^12,14^.

Antibiotic resistance genes (ARGs) that are most frequently detected in German urban wastewaters (*sul1, ermB, bla*_TEM_, *tetM*) as well as “intermediate and rare abundant”, which encode resistances to “Highest Priority Critically Important Antimicrobials” (*bla*_CTX-M-15_, *bla*_CTX-M-32_, *bla*_CMY-2_, *mcr-1*, *bla*_OXA-48_, *bla*_NDM-1_, *vanA*), were quantitatively amplified as previously published^13,70^. Furthermore, the facultative pathogenic bacteria *E. coli* (*yccT*), *K. pneumoniae* (*gltA*), *A. baumannii* (*secE*), *P. aeruginosa* (*ecfX*), and enterococci (23S rDNA) were quantified^13,70^. For qPCR analysis, a Bio-Rad Cycler CFX96 (CFX96 Touch Deep Well Real-Time PCR Detection System, Bio-Rad, Munich, Germany) and SYBR Green qPCR approach according to Hembach et al.^70^ and Jäger et al.^12^ were used. All samples were measured in technical triplicates. Cell equivalents were calculated according to Hembach et al.^70^ and normalized to 100 mL of water sample. In addition, a one-tailed nonparametric Mann–Whitney U test (Origin 8.1, OriginLab Corporation, Northampton, USA) was performed to identify the significance of the reduction in target genes during conventional and advanced wastewater treatments. Full primer sequences, qPCR reagent rations, thermal conditions and corresponding limit of detections (LODs) are listed in Supplemental Material (Tables S1, S2).

### Determination of antimicrobial residues

Water samples were analysed for 45 antibiotics and two metabolites (N-acetylsulfamethoxazole and anhydroerythromycin) by high-performance liquid chromatography (HPLC) coupled to tandem mass spectrometry (MSMS) after dilution and filtration through hydrophilic PTFE filters (Macherey–Nagel, Düren, Germany) as previously described^71^. The analysed antibiotics belong to the following substance classes: β-lactams (i.e. penicillins, cephalosporins and carbapenems), tetracyclines, fluoroquinolones, sulfonamides (as well as their synergist trimethoprim), macrolides including tylosin and spiramycin, lincosamides, glycopeptides, oxazolidinones, and nitroimidazoles. All analysed antibiotics, including their limit of quantification (LOQ), are given in Supplemental Material Table S3. The LOD of each individual analyte was one-third of the respective LOQ.

## Supplementary Information

Supplementary Information.
