# Supplementary material for: Antibiotic-resistant bacteria, antibiotic resistance genes, and antibiotic residues in wastewater from a poultry slaughterhouse after conventional and advanced treatments
Source: Sci Rep. 2021 Aug 17;11:16622. doi: 10.1038/s41598-021-96169-y (PMC8371126; doi:10.1038/s41598-021-96169-y)
Supplement: Supplementary file 1 — Supplementary Information. [file 41598_2021_96169_MOESM1_ESM.pdf]

Supplementary Material

Antibiotic-resistant bacteria, antibiotic resistance genes, and antibiotic residues in wastewater from a poultry slaughterhouse after conventional and advanced treatments

Mykhailo Savin*, Johannes Alexander, Gabriele Bierbaum, Jens Andre Hammerl, Norman Hembach, Thomas Schwartz, Ricarda Maria Schmithausen, Esther Sib, Alexander Voigt, Judith Kreyenschmidt

*corresponding author:

Dr. Mykhailo Savin, Institute for Hygiene and Public Health, Medical Faculty, University of Bonn, Venusberg-Campus 1, 53127 Bonn, Germany. Electronic address: m.savin@unibonn.de

Table S1 Temperature profile for every qPCR run, annealing temperature was the same for every primer used.

Temperature	Time	Reaction step	Cycle
90 °C	10 min	Initial denaturation and enzym-activation	x 1
90 °C	15 sec	Denaturation	x 40
60 °C	1 min	Annealing/Elongation	
60 – 90 °C	5 sec / 0,5 °C	Melt curve	x 1

PCR reagent ratio for each qPCR reaction volume was: 10 µL 2x Maxima Sybr Green Master Mix, 0.5 µL forward and 0.5 µL reverse primer (final concentration was 250 nM each), 8 µL nuclease free PCR water and 1 µL template DNA.

Table S2 Full primer sequences of investigated FPB and ARGs with corresponding calibration curve, efficiency and LOD.

target	primer sequence	calibration curve	amplicon	efficiency	R^2	LOD	reference strain	literature
**facultative pathogenic bacteria**								
enterococci	Fwd: AGAAATTCCAAACGAACTT G Rev: CAGTGCTCTACCTCCATCAT T	F(x)= -3.585x+35.283	93 bp	90.1 %	1.000	65	*E. faecium* DSM20477	(Frahm et al. 2003)
P. aeruginosa	Fwd: AGCGTTCGTCCTGCACAAGT Rev: TCCACCATGCTCAGGGAGAT	F(x)= -3.282x+35.276	81 bp	101.7 %	0.999	3	*P. aeruginosa* DSM1117	(Clifford et al. 2012)
K. pneumoniae	Fwd: ACGGCCGAATATGACGAATT C Rev: AGAGTGATCTGCTCATGAA	F(x)= -3.387x+38.844	68 bp	97.4 %	0.998	18	*K. pneumoniae* DSM30104	(Clifford et al. 2012)
A. baumannii	Fwd: GTTGTGGCTTTAGGTTTATTATACG Rev: AAGTTACTCGACGCAATTCG	F(x)= -3.380x+35.679	94 bp	97.6 %	1.000	31	*A. baumannii* DSM30007	(Clifford et al. 2012)
E. coli	Fwd: GCATCGTGACCACCTTGA Rev: CAGCGTGGTGGCAAAA	F(x)= -3.361x+35.797	59 bp	98.4 %	0.994	4	*E. coli* DSM1103	(Clifford et al. 2012)
**antibiotic resistance genes**								
*erm*B	Fwd: TGAATCGAGACTTGAGTGTGCAA Rev: GGATTCTACAAGCGTACCTT	F(x)=-3.328x+35.901	71 bp	100 %	1.000	16	*S. hyointestinalis* DSM20770	(Alexander et al. 2015)
*tet*M	Fwd:GGTTTCTCTTGGATACTTAAATC AATC Rev: CCAACCATAAATCCTTGTTCRC	F(x)=-3.424x+38.747	88 bp	95.9 %	0.998	4	*E. coli* DH5α	(Peak et al. 2007)
bla_{TEM}	Fwd: TTCCTGTTTTTGCTCACCCAG Rev: CTCAAGGATCTTACCGCTGTTG	F(x)=-3.303x+38.559	112 bp	100.8 %	0.999	80	*E. coli* pNORM	(Rocha et al. 2018)

*sul*1	Fwd: CGCACCGGAAACATCGCTGCAC Rev: TGAAGTTCCGCCGCAAGGCTCG	F(x) -3.387x+39.802	161 bp	97.6 %	0.999	80	*E. coli* pNORM	(Rocha et al. 2018)
bla$_{CMY-2}$	Fwd: CGTTAATCGCACCATCACC Rev: CGTCTTACTAACCGATCCTA GC	F(x)= -3.591x+34.026	172 bp	89.9 %	0.998	71	*K. pneumoniae* NRZ-01013	(Kurpiel and Hanson 2011)
*van*A	Fwd: TCTGCAATAGAGATAGCCG C Rev: GGAGTAGCTATCCCAGCAT T	F(x)= -3,541x+33,078	376 bp	91.6 %	1.000	43	*E. faecium* B7641	(Klein et al. 1998)
mcr-1	Fwd: GGGCCTGCGTATTTTAAGC G Rev: CATAGGCATTGCTGTGCGT C	F(x)=-3,386x+35,349	183 bp	97.4 %	0.999	8	*E. coli* NRZ-14408	(Hembach et al. 2017)
bla$_{NDM-1}$	Fwd: TTGGCCTTGCTGTCCTTG Rev: ACACCAGTGACAATATCACCG	F(x)= -3,293x+35,877	82 bp	101.2 %	0.999	66	*K. pneumoniae* ATCC BAA-2146	(Monteiro et al. 2012)
bla$_{CTX-M15}$	Fwd: CGCTTTGCGATGTGCAG Rev: ACCGCGATATCGTTGGT	F(x)= -3.504x+34.255	551 bp	92.9 %	1.000	93	*E. coli* pNORM	(Paterson et al. 2003, Rocha et al. 2018)
bla$_{CTX-M-32}$	Fwd: CGTCACGCTGTTGTTAGGA A Rev: CGCTCATCAGCACGATAAA G	F(x)= -3.517x+37.800	155 bp	92.5 %	1.000	235	*E. coli* pNORM	(Rocha et al. 2018)
bla$_{OXA-48}$	Fwd: TGTTTTTGGTGGCATCGAT Rev: GTAAMRATGCTTGGTTCGC	F(x)= -3.540x+36.913	177 bp	91.6 %	0.998	92	*K. pneumoniae* TGH Isolate 2	(Monteiro et al. 2012)

Table S3 List of all investigated antibiotics and their metabolites (limit of quantification, ng/L)

**β-Lactams**				
**Penicillins**				
Amoxicillin (50)	Ampicillin (200)	Penicillin G (50)	Cloxacillin (20)	Dicloxacillin (20)
Flucloxacillin (20)	Methicillin (10)	Mezlocillin (20)	Nafcillin (20)	Oxacillin (10)
Penicilin V (20)	Piperacillin (100)			
**Carbapenems**				
Meropenem (200)				
**Cephalosporines**				
Cefaclor (50)	Cefotaxime (50)	Ceftazidime (100)		
**Macrolides and lincosamides**				
Azithromycin (50)	Clarithromycin (50)	Clindamycin (20)	Erythromycin (20)	Anhydroerythromycin (20)
Roxithromycin (50)	Spiramycin (100)	Tylosin (50)		
**Tetracyclines**				
Chlortetracycline (200)	Doxycycline (200)	Oxytetracycline (200)	Tetracycline (200)	
**Fluoroquinolones**				
Ciprofloxacin (200)	Enrofloxacin (200)	Moxifloxacin (200)	Ofloxacin (200)	
**Sulfonamides**				
Sulfachlorpyridazine (50)	Sulfadiazine (100)	Sulfadimethoxine (50)	Sulfadimidine (20)	Sulfadoxine (50)
Sulfaethoxypyridazine (50)	Sulfamerazine (50)	Sulfamethoxazole (20)	N4-Acetylsulfamethoxazole (100)	
Sulfamethoxypyridazine (10)	Sulfathiazole (100)	Trimethoprim (20)		
**Others**				
Linezolid (100)	Vancomycin (100)	Metronidazole (100)		
